# Postoperative neurocognitive disorders in the elderly: how can we stop the harm? A literature review

**DOI:** 10.3389/fmed.2025.1525639

**Published:** 2025-03-06

**Authors:** Ling Ma, Huthaifa Jasem Jasem, Wan Jun Gu, Qi Zeng, Xin Wang, Xu Dan Liu

**Affiliations:** ^1^Department of Anesthesiology, Shenzhen Hospital of Integrated Traditional Chinese and Western Medicine, Shenzhen, China; ^2^Department of Anesthesiology, Shengjing Hospital of China Medical University, Shenyang, China; ^3^Labor Health and Occupational Disease Teaching and Research Office, School of Public Health, China Medical University, Shenyang, China

**Keywords:** neuroinflammation, neuroprotection, elderly, postoperative neurocognitive disorders, postoperative complications

## Abstract

Postoperative neurocognitive disorders (PND) represent a significant challenge affecting patients undergoing surgical procedures, particularly in the elderly population. These disorders can lead to profound impairments in cognitive function, impacting memory, attention, and overall quality of life. Despite ongoing research efforts to identify risk factors and improve management strategies, PND remains underdiagnosed and poorly understood, complicating postoperative recovery and rehabilitation. This review aims to explore the recent advancement in the literature about PND, focusing on the underlying mechanisms, risk factors, and potential therapeutic approaches. We highlight recent advancements in the understanding of neuroinflammation, and it is implications for novel therapies to prevent PND. By synthesizing the latest research, we hope to provide insights that could lead to improved outcomes for patients at risk for PND and foster a shift towards more effective preventive measures in such population.

## Graphical abstract

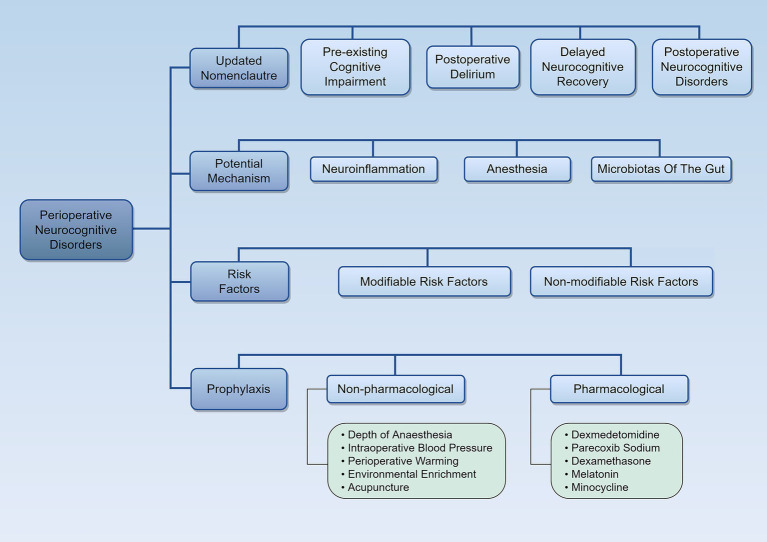

Graphical abstract of the review’s content.

## 1 Introduction

Postoperative neurocognitive disorders (PND) represent a growing, yet not well-understood threat to the surgical patients, especially those aging ≥65 years undergoing a major surgical procedure under general anesthesia (GA) (1). PND is a syndrome characterized by a decline from a preoperative state in multiple cognitive domains including attention, memory, language, emotion and executive functions following anesthesia and surgery (2). Since consensus about neurocognitive tests used to diagnose the syndrome is yet to be achieved, incidence can vary hugely across literature, with studies reporting as high as 50 to 60% (3–5), making it the most common postoperative complication in the elderly. PND results in a major social and economic burden to both the patients and their families, and to the health care providers. It causes an increased morbidity and mortality, worsening of present neurological and mental diseases, longer hospital stay and overall higher costs of health services (6). Since the emerging of this new entity of neurocognitive syndrome, many strategies have been investigated to alter the course of the cognitive decline, both pharmacologically and non-pharmacologically (7–9). This review aims to provide an insight into the recent updates on prevention and protection against PND. A summary of this review is shown in Graphical abstract.

## 2 Nomenclature

The lack of a unified and widely adopted terminology for postoperative cognitive complications has been a major issue in the literature, contributing significantly to the chaos in the existing literature. Authors and researchers have employed a wide variety of terms interchangeably, including postoperative cognitive impairment, postoperative cognitive dysfunction (POCD), perioperative cognitive disorder, and other related terms, often without clear distinctions or consistent definitions. This terminological inconsistency has created substantial challenges in several key areas including synthesizing research findings, comparing study results, establishing clear diagnostic criteria, and developing management guidelines.

**Figure 1:**
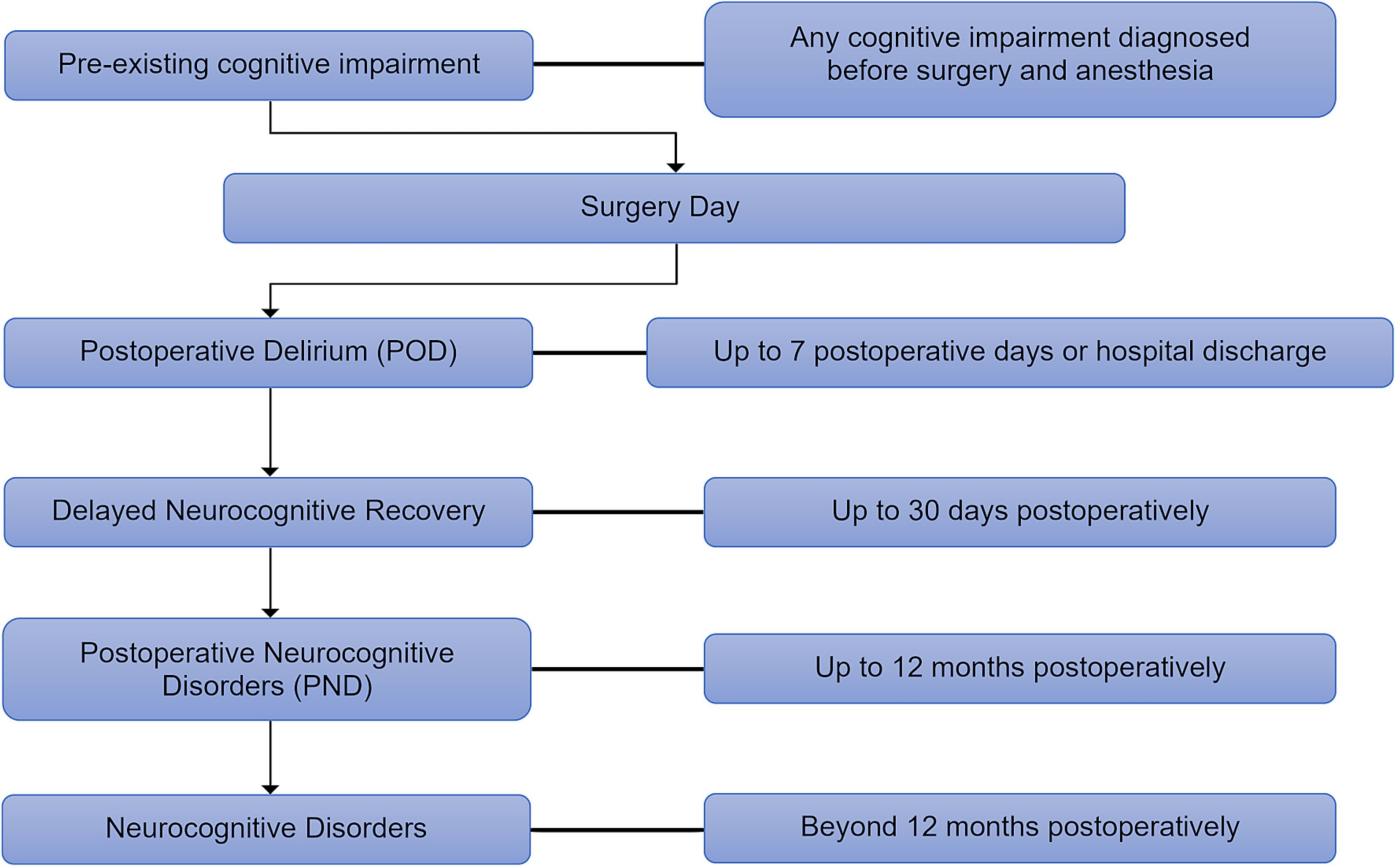
The updated nomenclature timeline of perioperative neurocognitive disorders. The new guideline classifies perioperative neurocognitive disorders (p-NCD) based on their onset and duration, outlining their progression from postoperative delirium (POD) (up to 7 days) to delayed neurocognitive recovery (up to 30 days), postoperative neurocognitive disorders (PND) (up to 12 months), and persistent neurocognitive disorders (beyond 12 months).

In response to this significant heterogeneity in the definitions and classifications of postoperative cognitive complications, recommendations were published in 2018 regarding the nomenclature of cognitive decline in surgical patients (10), sharing the updated terminology across six accredited journals (Figure 1). This landmark effort aimed to bring much-needed standardization to the field, improving communication and collaboration among researchers and clinicians.

A crucial aspect of this nomenclature is its temporal limitation; it applies only within the 12-month period following the surgical event and anesthesia. This time limitation is based on the understanding that the physiological and psychological effects of surgery and anesthesia are expected to dissipate within a year. Any cognitive decline diagnosed after this 12-month period should not retain the “postoperative” specifier. Such cases should instead be classified according to the established criteria for mild or major neurocognitive disorder (NCD) as defined for the general population, recognizing that these conditions may have different etiologies and require distinct diagnostic and management approaches. This distinction is crucial for ensuring accurate diagnosis, appropriate treatment, and meaningful research comparisons. The adoption of these standardized terms is essential for advancing our understanding of PND and for improving patient care.

## 3 Potential mechanisms

Despite decades of intensive research, the precise mechanisms underlying PND remain elusive (11–14). While a single, definitive pathway responsible for post-surgical cognitive changes has yet to be identified, substantial evidence points to PND as a multifactorial disorder, influenced by a complex interplay of factors that directly or indirectly impact brain function and cognitive performance (15–17). These factors can be broadly categorized into those related to neuroinflammation, the effects of anesthesia, and the influence of the gut microbiota.

### 3.1 Neuroinflammation

**Figure 2:**
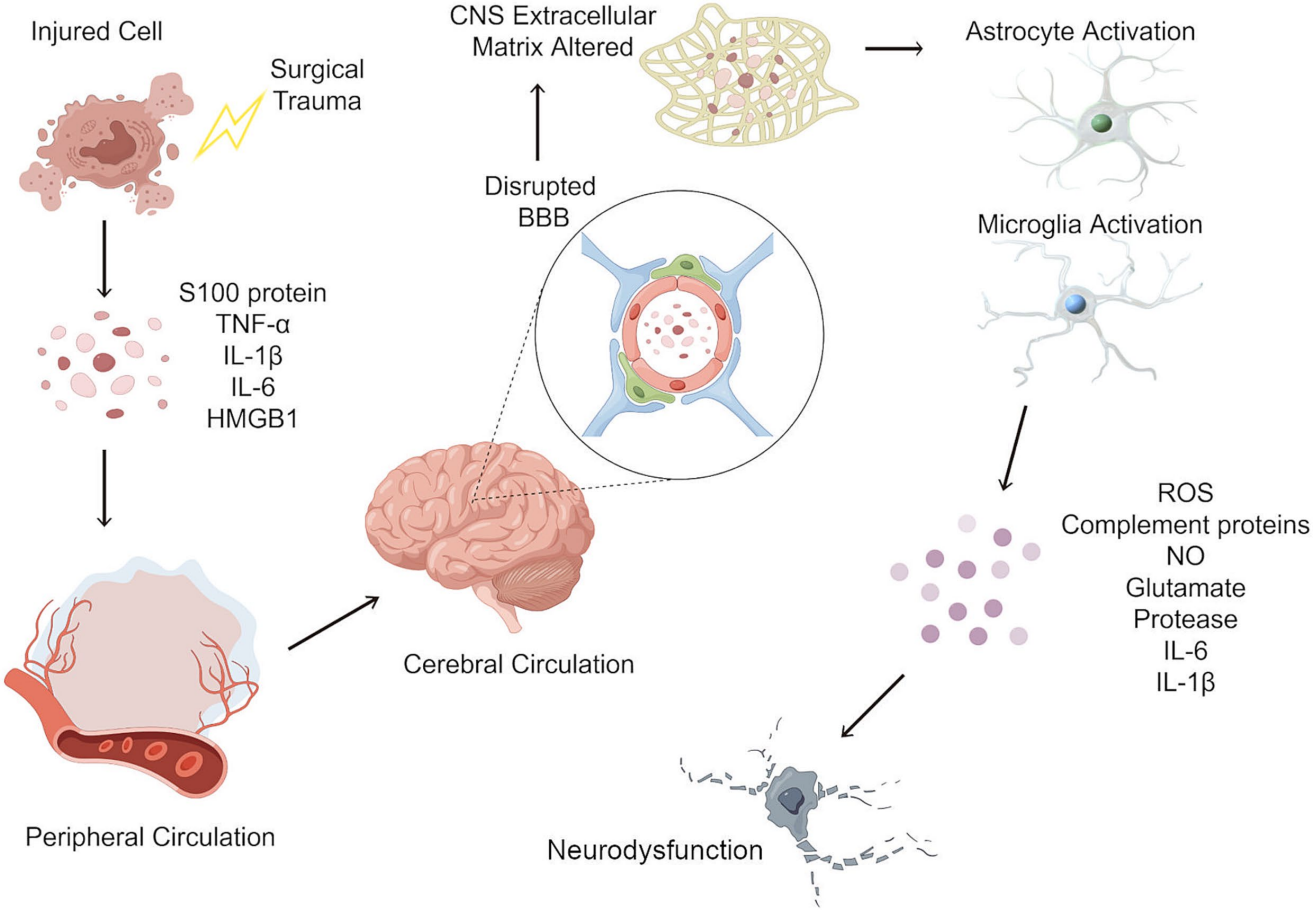
Neuroinflammatory cascade following surgical trauma and its role in postoperative neurocognitive disorders. Surgical trauma induces a systemic inflammatory response, leading to the release of proinflammatory cytokines and molecules such as IL-1β, IL-6, TNF-*α*, and HMGB1 into the bloodstream. These mediators enter the cerebral circulation, where they act upon endothelial cells of the blood–brain barrier (BBB), disrupting tight junctions and increasing permeability. The resulting uncontrolled influx of peripheral inflammatory molecules into the CNS contributes to neuroinflammation and oxidative stress. Within the CNS, proinflammatory cytokines activate resident immune cells, including astrocytes and microglia, initiating a pathological inflammatory response. Activated microglia and astrocytes release cytotoxic mediators, including glutamate, complement proteins, reactive oxygen species (ROS), and nitric oxide (NO). This excessive neuroinflammatory response leads to neuronal dysfunction, synaptic impairment, and neurotoxicity, which may result in transient or permanent changes to the local neuronal cells. IL-1β, Interleukin 1 beta; IL-6, Interleukin 6; TNF-α, tumor necrosis factor alpha; HMGB1, high mobility group box-1; CNS, central nervous system.

One of the most extensively studied hypotheses centers on the role of neuroinflammation in the development of PND (18), as illustrated in Figure 2. Surgical trauma induces a systemic inflammatory response, leading to the release of proinflammatory cytokines and molecules into the bloodstream. These mediators enter the cerebral circulation, where they act upon endothelial cells of the blood–brain barrier (BBB), disrupting tight junctions and increasing permeability. The resulting uncontrolled influx of peripheral inflammatory molecules into the CNS contributes to neuroinflammation and oxidative stress. Within the CNS, proinflammatory cytokines activate resident immune cells, including astrocytes and microglia, initiating a pathological inflammatory response. Activated microglia and astrocytes release cytotoxic mediators resulting in transient or permanent cognitive impairment, depending on the severity of neuroinflammation, the extent of neuronal damage, and the patient’s baseline cognitive reserve. In some cases, neuronal function may recover following the resolution of inflammation, while in others, prolonged neuroinflammation may lead to irreversible neuronal loss and long-term neurocognitive decline (15).

#### 3.1.1 Microglia: the CNS’s first responders

Microglia, the resident immune cells of the CNS, play a crucial role in maintaining CNS homeostasis. Similar to peripheral macrophages, microglia are responsible for clearing cellular debris and promoting neuronal survival (19). However, in the elderly, microglia often undergo age-related changes, including a shift towards a pro-inflammatory phenotype. This imbalance in the production of pro- and anti-inflammatory cytokines creates a neurotoxic environment within the CNS, contributing to neuronal damage and dysfunction (20). Further research is needed to understand the specific microglial alterations that contribute to PND and to identify potential therapeutic targets to modulate microglial activation.

#### 3.1.2 Astrocytes: guardians of neuronal health

Astrocytes, the most abundant glial cells in the CNS, are essential for maintaining neuronal health and function. They play a vital role in transporting essential nutrients, such as glucose and lactate, to neurons and regulating the extracellular environment by controlling glutamate levels (21). As brain ages, astrocytic functions deteriorate, leading to disruptions in energy metabolism and an accumulation of neurotoxic glutamate (22). This disruption in astrocytic function can further contribute to the neuroinflammatory processes implicated in PND. Investigating the specific mechanisms by which aging affects astrocytes and their contribution to PND is a crucial area for future research.

### 3.2 Role of anesthesia

The contribution of anesthesia to PND remains a subject of considerable debate. While some studies have shown a strong association between certain anesthetic agents and cognitive decline (23, 24), others have failed to establish a clear causal link (25, 26). This discrepancy may be due to several factors, including variations in study design, patient populations, and the specific anesthetic techniques employed.

#### 3.2.1 General versus non-general anesthesia

A key area of investigation focuses on the differential effects of GA versus non-GA (regional or local anesthesia) on PND. GA agents cross the BBB and suppress CNS activity, potentially contributing to neurocognitive decline. However, the extent and duration of these effects remain debated.

A meta-analysis by Gao et al. (27) examined seven randomized controlled trials (RCTs) involving 1,031 patients (526 in the GA group and 505 in the non-GA group) to assess the impact of anesthesia type on PND. The results showed that PND incidence was significantly higher in GA patients in the early postoperative period. On postoperative day one, three studies reported a nearly fourfold increase in PND risk in GA patients. By day three, two studies confirmed an increased risk, with GA patients experiencing twice the likelihood of PND. However, by day seven, five studies found no significant difference between groups, and at 3 months, two studies reported comparable PND incidence. These findings suggest that GA-related cognitive impairment is primarily transient, affecting patients in the immediate postoperative period but resolving over time.

#### 3.2.2 Total intravenous anesthesia versus inhalational anesthesia

Another area of focus involves comparing the effects of propofol-based total intravenous anesthesia (TIVA) versus inhalational anesthetics such as sevoflurane or isoflurane on PND in elderly patients. Pang et al. (28) conducted a meta-analysis of 15 RCTs involving 1,854 elderly patients undergoing non-cardiac surgery, evaluating PND incidence and postoperative cognitive status at different time points.

Their findings suggested that propofol anesthesia was associated with a lower risk of early PND. Between postoperative days 2 and 6, patients receiving propofol had a significantly lower incidence of PND compared to those receiving inhalational anesthesia. Additionally, Mini-Mental State Examination (MMSE) scores were higher in the propofol group, suggesting better postoperative cognitive function. The analysis also showed that propofol anesthesia was linked to lower levels of systemic inflammation, with reduced Interleukin-6 (IL-6) and Tumor Necrosis Factor-alpha (TNF-*α*) compared to inhalational agents.

### 3.3 Microbiotas of the gut

The gut microbiota, the complex community of microorganisms residing in the gastrointestinal tract (GIT), is now recognized as a significant modulator of various physiological processes, including immune responses, metabolic pathways, and even brain function (17, 29). Emerging evidence suggests that alterations in the gut microbiota may contribute to PND by influencing the gut-brain axis, the bidirectional communication pathway connecting the digestive system and the CNS (30–33).

Surgery and general anesthesia significantly alter gut microbiota composition through multiple mechanisms. Surgical stress and immune dysregulation disrupt gut barrier integrity, reducing beneficial bacteria while promoting pathogenic overgrowth (34). General anesthesia further alters microbial diversity and increases intestinal permeability, allowing bacterial metabolites like lipopolysaccharides to trigger neuroinflammation (35). Antibiotic prophylaxis exacerbates dysbiosis by reducing microbial diversity, increasing the risk of PND (36).

Microbiota-targeted interventions are being explored to mitigate PND by restoring gut microbial balance and reducing neuroinflammation. Probiotics, particularly strains like *Lactobacillus rhamnosus* and *Bifidobacterium longum*, have shown neuroprotective effects in animal models, though clinical evidence on their role in PND prevention remains limited (37, 38). Fecal Microbiota Transplantation (FMT), primarily used for Clostridioides difficile infections, has demonstrated cognitive benefits in neurological disorders, with animal studies suggesting its potential for PND prevention by improving microbiota composition and reducing inflammation (39, 40). Additionally, dietary interventions rich in fiber, polyphenols, and fermented foods may support a healthy gut-brain axis, while exercise and stress reduction have been linked to improved gut microbial composition and cognitive resilience (41).

Recent studies highlight the role of gut microbiota in PND development, with postoperative shifts in microbial composition linked to neuroinflammation and cognitive decline. Zhang et al. (30–33) found that reduced anti-inflammatory gut bacteria and increased Bacteroides levels were associated with PND in mice, suggesting gut microbiota modulation as a potential preventive strategy. Similarly, Zhang et al. (30–33) identified a correlation between higher *Parabacteroides distasonis* levels and POD in elderly surgical patients. While promising, further clinical studies are needed to validate the long-term efficacy of these approaches in improving postoperative cognitive outcomes.

## 4 Risk factors

The development of PND is not a universal outcome following surgery under GA. Identifying key risk factors is crucial, particularly in the elderly, who are more vulnerable due to age-related neurological and physiological changes.

The first international study of postoperative cognitive dysfunction (ISPOCD 1) identified various patient-related and non-patient-related factors contributing to PND. These risk factors can be categorized as modifiable and non-modifiable, as outlined in Table 1 (6, 9, 42–45).

**Table 1:** Modifiable and non-modifiable risk factors.

Modifiable risk factors	Non-modifiable risk factors
Anesthetic type	Old age
Anesthesia depth	Male sex
Intraoperative hypotension	Surgery type
Postoperative pain	Surgery duration
Sleep quality	Frailty
	Pre-existing cognitive decline
	ASA III or above

ASA, American society of anesthesiologist.

Among non-modifiable risk factors, advanced age, frailty, and pre-existing cognitive impairment significantly increase susceptibility to PND. Older adults have reduced cognitive reserve, making them more prone to POD and long-term cognitive decline. Other factors such as male sex, surgery type and duration, and a higher ASA classification (≥III) also correlate with increased neurocognitive vulnerability.

In contrast, modifiable risk factors provide opportunities for intervention. Optimizing anesthetic protocols, maintaining hemodynamic stability, ensuring effective pain management, and promoting adequate sleep can help reduce the risk of PND. Addressing these factors is particularly important for elderly patients, as they are more susceptible to perioperative neurocognitive decline.

Given the rising number of surgeries in older adults, a targeted approach focusing on modifiable risk factors is essential for improving postoperative cognitive outcomes.

## 5 PND prevention

Despite the many attempts to ameliorate the harm of PND, currently, there is no definitive evidence-based management plan for patients who develop postoperative cognitive impairment. This may be due to variations in defining, testing, and diagnosing this disorder across the literature. The current focus of many RCTs is examining various neuroprotective measures to stop the development of PND (9, 27, 46, 47).

### 5.1 Non-pharmacological interventions

#### 5.1.1 Depth of anesthesia

Depth of anesthesia (DoA), the level of unconsciousness during GA, is achieved through anesthetic agents that suppress CNS activity, reducing responsiveness to stimuli. Traditionally, DoA assessment relied on subjective methods such as monitoring vital signs and patient movement, which are prone to bias and inaccuracy, sometimes leading to intraoperative awareness and psychological distress, including Posttraumatic stress disorder (48). This underscores the need for objective and precise monitoring, particularly in elderly patients, who are more sensitive to anesthetics and require lower doses to achieve the same level of unconsciousness. The development of EEG-based monitoring, such as the BIS monitor, has significantly improved anesthesia precision and safety by providing a numerical value (0–100) that correlates with anesthesia depth (49, 50). Maintaining an optimal BIS range of 40–60 during surgery balances anesthesia with reduced intraoperative and postoperative complications, including a lower risk of awareness (51), attenuated inflammatory responses (52) and a decreased incidence of PND.

Numerous studies suggest that excessive anesthesia depth is more strongly associated with PND than insufficient anesthesia. A sub-analysis of the BALANCED study (53) found that light anesthesia (BIS 50) resulted in a lower incidence of POD (19% vs. 28%) compared to deep anesthesia (BIS 35). This is particularly significant in elderly patients, as deeper anesthesia has been linked to greater neurocognitive impairment due to increased anesthetic sensitivity. Similarly, a Cochrane review (54) and a meta-analysis (55) support BIS-guided light anesthesia as a strategy to reduce POD, reinforcing its neuroprotective role in the elderly.

However, some studies present conflicting findings. The ENGAGES trial (56) recorded only a mild reduction in POD incidence with BIS monitoring (26% vs. 23%). Likewise, a meta-analysis by Wang et al. (57) found no consistent correlation between light anesthesia (BIS 50) and lower POD incidence compared to deeper anesthesia (BIS 35). These discrepancies suggest that while BIS monitoring optimizes anesthesia depth, its direct impact on PND prevention remains debated.

Despite this, BIS monitoring remains particularly valuable in elderly patients, who face a higher risk of PND, intraoperative awareness, and prolonged recovery. Given age-related changes in drug metabolism and anesthetic sensitivity, BIS helps prevent both under-sedation and over-sedation, minimizing neurocognitive complications. Integrating BIS into anesthesia protocols for older adults offers benefits beyond PND prevention, including reduced anesthetic exposure and enhanced postoperative recovery.

#### 5.1.2 Intraoperative blood pressure

Intraoperative hypotension (IOH), defined as a systolic blood pressure (SBP) below 90 mmHg or a mean arterial pressure (MAP) below 60 mmHg, is a common complication during surgical procedures and anesthesia (58). Various factors contribute to IOH, including the effects of anesthetic agents, hypovolemia, and patient positioning (59). IOH significantly compromises perfusion to vital organs, and if left untreated, can lead to serious complications, including myocardial infarction (MI), cerebrovascular events, and acute kidney injury (AKI) (60, 61). Given the age-related decline in cerebrovascular autoregulation, elderly patients are particularly susceptible to the adverse effects of IOH. Reduced vascular elasticity, impaired baroreceptor sensitivity, and pre-existing comorbidities such as hypertension increase their vulnerability to cerebral hypoperfusion, potentially exacerbating postoperative neurocognitive impairment.

Researchers have hypothesized a link between IOH and PND, particularly in elderly patients, where compromised cerebral perfusion may increase POD and long-term cognitive decline (62). Some studies support this association, particularly when IOH episodes are prolonged. For instance, Mohr et al. (63) found that IOH episodes lasting more than 2 min were associated with a higher incidence of POD following cardiac surgery. Additionally, Krzych et al. (64) proposed targeting IOH as a modifiable risk factor for PND, reinforcing the importance of maintaining stable blood pressure during surgery. However, many of these studies are retrospective, limiting their ability to establish causality.

Conversely, other studies have failed to demonstrate a significant correlation between IOH and subsequent neurological dysfunction. Langer et al. (65) compared non-cardiac surgical patients with targeted higher MAP maintenance to a control group without targeted MAP management, finding no significant difference in PND incidence between the two groups. Similarly, a post-hoc analysis of the DECADE trial reported a negative association between IOH and POD (66). These conflicting findings suggest that the relationship between IOH and PND remains complex, potentially influenced by patient-specific factors such as cerebral autoregulation, pre-existing cognitive impairment, and individual susceptibility to hypoperfusion.

While current evidence does not definitively establish a causal link between IOH and PND, maintaining adequate intraoperative blood pressure is particularly critical in elderly patients. Given their reduced physiological reserve, strategies should focus on personalized blood pressure management, avoiding both hypotension, which may exacerbate cerebral ischemia, and excessive hypertension, which could increase the risk of cerebrovascular complications.

#### 5.1.3 Perioperative warming

Hypothermia, defined as a core body temperature below 36°C, is a common occurrence in surgical patients (67). Several factors contribute to intraoperative hypothermia, including the effects of anesthesia, cool operating room temperatures, and heat loss during surgical procedures (68). Hypothermia is associated with various adverse postoperative outcomes, including coagulopathy, delayed wound healing, and increased risk of surgical site infections (SSIs) (69). These complications highlight the importance of effective perioperative warming strategies, particularly in elderly patients, who are more vulnerable to hypothermia due to impaired thermoregulation, reduced metabolic heat production, and altered vasomotor responses.

Perioperative warming techniques can be broadly categorized as active or passive. Active methods, such as forced-air warming systems and heated intravenous fluids, actively raise core body temperature, while passive methods rely on thermal insulation to minimize heat loss (70). Maintaining normothermia perioperatively has been consistently linked to reduced rates of wound infections, shivering, intraoperative blood loss, and even mortality (71, 72). Given that elderly patients experience greater difficulty in maintaining core body temperature, proactive temperature management is particularly crucial in reducing their perioperative risks.

However, the relationship between perioperative temperature and neurocognitive outcomes remains complex, particularly in the elderly. While normothermia is generally preferred, some studies suggest that mild hypothermia (34–35°C) may have neuroprotective effects in certain high-risk surgical contexts. RCTs have indicated that inducing mild hypothermia can reduce brain injury following aortic dissection repair and improve cognitive outcomes after cardiopulmonary bypass (CPB) (73, 74). These findings suggest that targeted temperature modulation could potentially benefit older adults undergoing specific high-risk procedures. Conversely, other studies have demonstrated a positive correlation between intraoperative hypothermia and the development of POD (75–77).

Elderly patients are already at increased risk for PND, and intraoperative hypothermia may exacerbate neurocognitive dysfunction by impairing cerebral autoregulation and reducing metabolic activity in the brain. However, inconsistencies in the literature regarding the impact of hypothermia on PND may be due to variations in surgical procedures, patient demographics, and differences in PND diagnostic criteria.

#### 5.1.4 Enriched environment

An Enriched Environment (EE) consists of cognitive, sensory, and social stimuli that enhance brain function. EE has been shown to improve memory, learning, attention, mood, and overall cognitive resilience (8, 78). The mechanisms underlying EE’s benefits include increased neuroplasticity, synaptogenesis, and neurogenesis, which may be particularly valuable in mitigating age-related cognitive decline and neurodegenerative diseases.

Numerous animal studies highlight EE’s cognitive benefits in aging. Speisman et al. (79) demonstrated that aged rats housed in EE exhibited better cognitive functions potentially via enhanced hippocampal neurogenesis and modulating neuroimmune cytokine signaling. Similarly, Mate et al. (80) found that EE reduced tau pathology and neurodegeneration, suggesting a potential neuroprotective role against age-related cognitive impairment. Speisman et al. (81) reported that EE improved synaptic plasticity and reduced oxidative stress in aging rodents, reinforcing its role in preserving cognitive function. These findings suggest that EE may slow cognitive decline and could be particularly relevant for elderly populations at risk of PND, dementia, or postoperative cognitive dysfunction.

While much of the clinical research on EE has focused on early development (82, 83), an increasing number of studies suggest its potential cognitive benefits in aging. For instance, one meta-analysis found that virtual reality-based exergames, an enriched and interactive form of cognitive and physical stimulation, significantly improve overall cognitive function, memory, and depressive outcomes in older adults, with greater effects observed in interventions of longer duration (84). Similarly, a randomized trial showed physical, cognitive, and combined training enhance cognition differently—physical training sustained concentration gains, cognitive training improved cognitive speed over time, and combined training had immediate and lasting benefits (85). These findings highlight the diverse yet complementary effects of enriched interventions in aging.

In older adults, these mechanisms could be particularly beneficial in mitigating PND and long-term postoperative cognitive dysfunction. However, clinical trials specifically targeting EE interventions in the elderly remain limited. While the evidence for EE’s cognitive benefits is compelling, further research is needed to optimize EE protocols for elderly individuals and assess their long-term effects on postoperative cognitive function.

#### 5.1.5 Acupuncture

Acupuncture, a therapeutic modality originating from Traditional Chinese Medicine (TCM), involves the insertion of thin needles into specific points on the body. While its precise mechanisms remain under investigation, recent studies suggest that acupuncture modulates neuronal activity, connective tissue, and muscle fibers (86, 87). These effects may influence neurotransmission, inflammation, and cerebral blood flow, factors that are particularly relevant to cognitive health in elderly patients, who are more susceptible to neuroinflammation and vascular impairment.

Beyond its well-established role in pain management (88, 89) and treatment of various medical conditions (90, 91), acupuncture has gained interest as a potential non-pharmacological intervention for cognitive enhancement. Emerging research suggests that acupuncture may positively influence memory, attention, and executive function, with applications in neurodegenerative and vascular conditions such as Alzheimer’s disease (92), Parkinson’s disease (93), post-stroke cognitive impairment (94), and vascular dementia (95). Given the age-related decline in cognitive function and increased risk of PND in the elderly, acupuncture could serve as a potential adjunct therapy to mitigate cognitive impairment following surgery.

In the postoperative period, acupuncture presents a cost-effective and low-risk intervention for reducing PND in older adults, a population particularly vulnerable to surgery-related cognitive decline. A review by Ho et al. (96) examined trials from 2009 to 2018, encompassing both clinical and preclinical studies. While the quality of evidence varied, findings indicated that acupuncture reduced inflammatory biomarkers, including S100β, Neuron-Specific Enolase (NSE), IL-6, and TNF-*α*, compared to controls. Given that neuroinflammation and oxidative stress play key roles in PND pathogenesis, these findings suggest a potential neuroprotective mechanism for acupuncture in surgical patients, particularly the elderly.

### 5.2 Pharmacological interventions

#### 5.2.1 Dexmedetomidine

Dexmedetomidine (DEX), a selective alpha-2 adrenergic agonist, is widely used as a sedative and anesthetic adjunct (97). Unlike traditional sedatives, DEX modulates sympathetic outflow, reducing norepinephrine release to induce sedation while preserving respiratory function (98).

Beyond its sedative and analgesic effects, DEX exerts anti-inflammatory properties. Preclinical studies demonstrate its ability to attenuate systemic inflammation. Zhang et al. (30) found that DEX reduced sepsis-induced organ injury by increasing IL-10 and nuclear receptor 77, while suppressing TNF-*α* and IL-1β. Similarly, Gao et al. (99) reported reduced neuroinflammation and preserved white matter in spinal cord-injured rats, effects reversed by α2-adrenergic receptor antagonism. Clinical evidence supports these findings; a sub-analysis of the DESIRE trial showed significantly lower C-reactive protein (CRP) and procalcitonin levels in DEX-treated sepsis patients (100).

These anti-inflammatory properties have led to investigations into DEX’s role in preventing PND, particularly in elderly surgical patients. Shin et al. (47) compared DEX-based vs. propofol-based sedation in elderly patients undergoing lower limb surgery, reporting a significantly lower incidence of POD in the DEX group, though with lower MAP and heart rate (HR) in the post-anesthesia care unit (PACU). Similarly, Ge et al. (101) found that DEX infusion improved cognitive function post-carotid endarterectomy (CEA), with higher MMSE and MoCA scores and reduced IL-6 and TNF-*α* levels in the first 72 h. While Brain-Derived Neurotrophic Factor levels were initially similar across groups, only the placebo group returned to baseline after 24 h. Further supporting this, Tang et al. (102) demonstrated that sufentanil plus DEX reduced POD incidence and severity after thoracoscopic-laparoscopic esophagectomy (TLE), accompanied by lower IL-6 and TNF-α, and higher IL-10 at 24 h postoperatively.

DEX’s mechanism, anti-inflammatory properties, and favorable safety profile make it a promising agent for PND prevention, particularly in the elderly and those with pre-existing cognitive impairment. Future research should refine optimal dosing strategies to maximize cognitive benefits while mitigating hemodynamic effects.

#### 5.2.2 Parecoxib sodium

Parecoxib sodium, a selective cyclooxygenase-2 (COX-2) inhibitor, is a potent analgesic and anti-inflammatory agent widely used in perioperative care (103). As the only injectable COX-2 inhibitor, it undergoes rapid hepatic metabolism to its active form, valdecoxib, and is typically administered intravenously or intramuscularly for acute pain management. Parecoxib sodium’s COX-2 selectivity provides effective pain relief while minimizing gastrointestinal and renal complications compared to non-selective NSAIDs (104).

Surgical stress-induced systemic inflammation is implicated in PND pathogenesis (105). Given parecoxib sodium’s anti-inflammatory properties, studies have investigated its neuroprotective effects in elderly surgical patients. Mu et al. (106) conducted a multicenter, double-blind RCT in elderly orthopedic patients under spinal anesthesia, finding that PND incidence decreased from 11 to 6.2% when parecoxib sodium was added to a postoperative morphine regimen. Similarly, Zhu et al. (107) examined elderly knee arthroplasty patients, reporting lower PND incidence at 1 week (16.7% vs. 33.9%), improved POP relief within the first 12 h, and reduced IL-1β, IL-6, and TNF-*α* plasma levels. However, differences in 3-month PND incidence and CRP levels were not statistically significant.

Parecoxib sodium offers potent analgesia, reduced opioid reliance, and a favorable safety profile in the perioperative setting. While its neuroprotective role in PND prevention remains promising, further high-quality trials are needed to establish definitive benefits and optimize perioperative anti-inflammatory strategies for elderly surgical patients at risk of PND.

#### 5.2.3 Dexamethasone

Dexamethasone, a synthetic glucocorticoid, is widely used in perioperative care due to its anti-inflammatory, immunomodulatory, analgesic, antiemetic, and anti-shivering properties (108–111). These effects contribute to improved postoperative recovery by reducing inflammation, nausea, pain, and thermoregulatory disturbances, making dexamethasone a valuable adjunct in multimodal anesthesia protocols (112).

Dexamethasone’s anti-inflammatory and neuroprotective properties have led to increasing interest in its potential role in reducing PND. Inflammation is a key contributor to PND pathogenesis, and glucocorticoids like dexamethasone may mitigate surgery-induced neuroinflammation, thereby improving postoperative cognitive outcomes. Several studies have examined dexamethasone’s impact on PND and POD in elderly surgical patients. Huang et al. (113) found that a single 10 mg dose of dexamethasone significantly reduced POD incidence compared to placebo in elderly patients undergoing intertrochanteric fracture surgery. Similarly, Kluger et al. (114) reported that a 20 mg dose reduced PND severity scores in hip fracture patients, though it did not significantly alter overall PND incidence.

These findings suggest that while dexamethasone may not completely prevent PND, it could help reduce its severity, potentially improving functional recovery and quality of life in elderly surgical patients. The mechanisms underlying dexamethasone’s neuroprotective effects remain an area of active investigation. Proposed pathways include suppression of pro-inflammatory cytokines (IL-6, TNF-*α*, IL-1β), reduction of blood–brain barrier dysfunction, and attenuation of neuroinflammation-induced oxidative stress (115, 116).

Dexamethasone’s broad-spectrum perioperative benefits, including its anti-inflammatory, analgesic, and antiemetic effects, make it a strong candidate for inclusion in perioperative anesthetic plans. Given its favorable safety profile with short-term use, dexamethasone represents a promising intervention for reducing PND risk. However, future studies should focus on determining the optimal dosage, particularly in elderly patients, to maximize its neuroprotective benefits while minimizing potential side effects.

#### 5.2.4 Melatonin

Melatonin, a pineal gland hormone, regulates the sleep–wake cycle and influences mood, immune function, and cognitive health through its widespread CNS and peripheral receptor distribution including the retina, GIT, and immune cells (117, 118). Exogenous melatonin has demonstrated benefits in insomnia, depression, anxiety, and migraines, with evidence suggesting neuroprotective effects in Alzheimer’s and Parkinson’s disease (119–121).

Sleep disturbances are common in surgical patients, with preoperative insomnia rates up to 79% (122). Melatonin supplementation improves sleep quality, reduces anxiety, and enhances recovery (123, 124). Its antioxidant and anti-inflammatory properties may also protect against PND (125, 126). A meta-analysis indicated melatonin reduces PND incidence, though results vary (127). For Elbakry et al. (128) found that 5 mg oral melatonin preoperatively reduced POD in elderly colorectal surgery patients, whereas Ford et al. (129) observed no effect with 3 mg in cardiac surgery, suggesting dose and surgical context may influence efficacy.

Melatonin shows potential for PND prevention, particularly in elderly patients, by addressing sleep disturbances and reducing neuroinflammation. Further research should optimize dosing and administration timing to enhance its perioperative neuroprotective benefits.

#### 5.2.5 Minocycline

Minocycline, a semisynthetic tetracycline antibiotic, is used for bacterial infections and has shown potential in treating chronic pain, epilepsy, and diabetic neuropathy due to its anti-inflammatory, anti-apoptotic, and immunomodulatory properties (130–132). Its lipophilic nature allows it to cross the BBB, suggesting neuroprotective effects (133, 134).

Animal studies indicate that minocycline regulates microglia, astrocytes, and neurons, inhibiting microglial activation and reducing pro-inflammatory cytokines such as TNF-*α*, IL-1β, and IL-6 (135, 136). Li et al. (137) demonstrated that older rats exposed to isoflurane anesthesia exhibited lower neuroinflammatory marker levels with minocycline treatment. However, some studies caution that while minocycline initially inhibits microglial activation, delayed microgliosis may negatively affect the hippocampus, potentially impairing long-term cognitive function (138).

Regarding PND, Liang et al. (139) suggested minocycline may mitigate anesthetic-induced cognitive impairment by protecting against sevoflurane-induced neuroinflammation. However, an RCT conducted by Takazawa et al. (140) in elderly knee arthroplasty patients found no significant difference in PND incidence or postoperative pain between minocycline and control groups. These conflicting results highlight the uncertain clinical efficacy of minocycline in perioperative neuroprotection.

While preclinical studies support minocycline’s neuroprotective and anti-inflammatory properties, clinical evidence remains inconclusive. Further rigorous trials are needed to clarify its efficacy in preventing PND, particularly in elderly surgical patients. Understanding optimal dosing, treatment duration, and patient selection will be essential in determining minocycline’s role in perioperative neuroprotection.

## 6 Conclusion

PND poses a significant threat to elderly patients, impacting their quality of life and placing a substantial burden on healthcare systems. The current lack of a standardized approach to diagnosis, assessment, and management, coupled with marked heterogeneity in research methodologies, hinders progress. Future research should prioritize the development of robust tools for quantifying PND risk factors and identifying specific biomarkers. Equally important is the creation of evidence-based management plans to effectively address PND in affected patients, thereby improving post-surgical outcomes and reducing the overall healthcare burden.
